# Promoting weaning practices and growth of Egyptian infants by using communication for behavioral development approach

**DOI:** 10.1186/s12887-022-03741-0

**Published:** 2022-12-01

**Authors:** Ammal M. Metwally, Sara F. Sallam, Mohamed A. Abdel Mawla, Khadija M. Alian, Ghada A. Abdel-Latif, Hasanin M. Hasanin, Ayat N. Kamal, Carine Hanna, Salwa M. El Shebini, Nihad H. Ahmed, Hoda B. Mabrok, Maha H. Mahmoud, Ahmed S. Ismail, Samia A. W. Boseila, Inas R. El-Alameey, Nermine N. Mahfouz, Fatma A. Shaaban, Nihad A. Ibrahim, Nayera E. Hassan, Sahar A. El-Masry, Maie M. Naga, Aya Khalil

**Affiliations:** 1 Community Medicine Research Department, Medical Research and Clinical Studies Institute, National Research Centre, Dokki, Cairo, 60014618 Egypt; 2 Child Health Department, Medical Research and Clinical Studies Institute, National Research Centre, Dokki, Cairo, 60014618 Egypt; 3 Pediatrics Department, Medical Research and Clinical Studies Institute, National Research Centre, Dokki, Cairo, 60014618 Egypt; 4 Biological Anthropology Department, Medical Research and Clinical Studies Institute, National Research Centre, Dokki, Cairo, 60014618 Egypt; 5 Nutrition and Food Science Department, Food Technology and Nutrition Research Institute, National Research Centre, Dokki, Cairo, 60014618 Egypt; 6 Faculty of Applied Medical Sciences, Clinical Nutrition Department, Taibahu University, Almadina almunawara, Saudi Arabia

**Keywords:** Communication for behavioral development (C4D), Indicators of complementary feeding practices, Dietary diversity

## Abstract

**Background:**

Access to various affordable and nutritious foods is considered a challenging factor for households with limited resources affecting the proper weaning practices. In order to motivate communities to adhere to the right and proper weaning practices, the social aspect should be considered through close communication with the targeted communities. This study aimed to evaluate how impactful the use of the principles of Communication for Development (C4D) that respect parents’ beliefs and their cultural norms is in improving the weaning practices and growth of infants in an Egyptian village.

**Methods:**

An interventional three-phase study was conducted for three years. The intervention targeted 464 mothers of infants up to 2 years of age. C4D interventions encouraged each mother to provide her baby with nutritious and varied options through age-appropriate introduction and diversification of nutrient-rich complementary foods under the slogan “ enjoy meals like a baby”. The effectiveness of the approach was measured by five essential weaning practices: Introduction of solid, semi-solid, or soft foods, Minimum dietary diversity, minimum meal frequency, Minimum acceptable diet, and consumption of iron-rich foods.

**Results:**

There was marked and significant improvement in the awareness and of the majority of the weaning practices’ indicators as a result of the interventions. This was noticed for the timely introduction of complementary foods which increased from 36.7% to 82.0%, the minimum meal frequency indicator (3–5) which increased from 25.3% to 67.3%, iron-rich or fortified food (68.0% to 82%) as well as a regular checkup for baby health at the health unit (71.3%). Indicators that were improved but failed to achieve the target were the “Minimum Dietary Diversity” (reached 32%) and the minimum acceptable diet (reached 22.0%). A significant effect on linear growth especially for females is evidenced by the remarkable decrease in wasting (from 31.5% to 11.1%) and obesity (from 12.0% to 0%) associated with a considerable decrease in underweight (from 40% to 16.7%).

**Conclusion:**

Targeting caregivers through the C4D approach have succeeded in providing them with the support required for the provision of adequate nutrition for their infants that had significantly marked improvement in growth indices of their infants.

**Supplementary Information:**

The online version contains supplementary material available at 10.1186/s12887-022-03741-0.

## Background

Appropriate nutrition during the 1,000-days beginning with the start of a woman’s pregnancy and her infant’s completing 24 months, is critical to her baby’s future health, and wellbeing [1–3]. According to the international and national experiences and recommendations, infants should be exclusively breastfed for the first 6 months of life, followed by proper weaning [4–7], so that proper nutrition could be achieved to prevent almost one-fifth of under-five mortality in developing countries [8].

WHO, 2018 recommends that achievement of proper weaning needs a timely introduction of adequate nutritious complementary food that is adequate in amount, frequency, consistency, and variety to address the nutritional needs of the growing infant at 6 months of age, along with sustained breastfeeding, up to 2 years of age and beyond [4–10]. In fact, receiving nutritionally adequate and safe complementary foods is limited globally; less than a fourth of infants 6– < 24 months of age meet the criteria of appropriate dietary diversity and feeding frequency according to their ages [11].

Different countries may have different approaches to weaning according to the country's culture [5]. Mothers’ own values and beliefs, as well as those of their society and the supporting networks such as fathers, grandmothers, and medical staff, always act as controlling factors for their weaning practices. Meanwhile, what is consistent is that weaning in Arab countries is considered problematic [12] and information gaps exist in relation to how, when weaning takes place and the effect of culture on weaning practices [13]. Egyptian studies revealed that nearly two-thirds of mothers wean their babies before the age of 6 months [6, 14, 15]. Furthermore, previous studies from the Middle East that were done in Kuwait [16], Saudi Arabia [17], Lebanon [18], Turkey [19], and The United Arab Emirates [20], reported different high rates of early weaning starting at the age of 3 months onwards with a wide range of rates (from 30.4% in Kuwait to 65% in The United Arab Emirates).

In order to motivate communities to adhere to the right and proper weaning practices, the social aspect should be considered through close communication with the targeted communities. This could be achieved through applying the principles of Communication for Development (C4D). Through C4D, understanding parents’ beliefs and their cultural norms could be done allowing communicating with them and facilitating their change at and for the best [21, 22].

This study aimed at investigating the effectiveness of the C4D approach to provide nutritional education interventions over 2 years duration on improving weaning practices of mothers/parents/caregivers of infants 6- < 24 months and improving the growth of their infants 18–36 months of age. Through the C4D approach, the interventions aimed at encouraging each mother to “feed her baby like a baby” meaning providing her baby nutritious and varied options through age-appropriate introduction and diversification of nutrient-rich complementary foods under the slogan “Enjoy Meals Like a Baby”. Five core weaning indicators were used to detect the effectiveness of CAD approach: Introduction of solid, semi-solid or soft foods, Minimum dietary diversity, Minimum meal frequency, Minimum acceptable diet and Consumption of iron-rich foods.

## Methodology

### Study design

The study was an interventional evaluation study with pre-and post-interventional comparison, conducted over three years’ duration starting in Jan. 2017 and ending in Jan. 2020. A quasi-experimental design with random selection and post-test only control design was done.

### Study setting

Two villages were targeted in this study as intervention and control villages. Both the intervention and the control villages belong to El Mahala district, in El Gharbyia governorate. The intervention village name is El Othmanyia village and the control one is Nemra el Basal village. Both villages have comparable socioeconomic characteristics but in different local village unit (one in the east and the other in the west of El Mahallah El Kubra district) to prevent possible contamination so that any real impact could be assessed. Both of them have powerful and active well-organized community-based associations (CBAs) namely: Charitable Patients’ Association in each village (CPA).

### Phases of the study

The study was conducted over three phases; phase I was formative assessment research, phase II was the intervention phase, and phase III which was the evaluation for measuring the outcome of the interventions as pre and post intervention comparison.

### Study participants

#### Study participants targeted for interventions

The targeted participants who were engaged in the interventions were 464 women in the age range 20–30 years who were mothers of infants 0–24 months; 294 of them were mothers of infants aged 6- < 24 months, including both who breastfed their infants and who did not. All targeted participants were married housewives who completed their preparatory level of education and have on average 3 children. The longitudinal interventions followed over 464 mothers from giving birth throughout their infant’s second year of life.

According to our listed inclusion criteria, the exclusion criteria included mothers who were younger than 20 years or older than 40 years or those who were mothers of infants above 24 months of age prior to the time of starting the intervention. For mothers who did not complete the four sessions within one year of the interventions, their initial data were discarded and they were excluded from being evaluated.

#### Study participants targeted for assessment and evaluation phases

The sample size was calculated for the study participants that were targeted during the assessment and evaluation according to Fleiss and his colleagues, 2003 and Newcomb, 1998 [23, 24]. A sample size of 141 houses produced a two-sided 90% confidence interval with a width equal to 0.100 when the sample proportion of early weaning is 0.870 [25] which was rounded to 150 households. The basis for sample size calculation during the assessment and evaluation phases was as follow:

The targeted village has four blocks (administrative sections determined by the Ministry of Health of Egypt), and each block was served by two social workers (one health care worker of the rural village health unit and one village promoter of the community-based association. To have a representative sample covering each block 50 eligible mothers were randomly selected by systematic random sample out of an average of 87 eligible mothers per block (25 eligible mothers per social worker).

Of 349 mothers who fulfilled the eligibility criteria for the study, the completed data analyzed according to the target group were as follow; 200 mothers for infants 0–24 months for the awareness indicators (50 mothers of infants 0–6 months out of 170 targeted mothers and 150 mothers for infants > 6–24 months out of 221 targeted mothers for practices indicators). The study also included 100 mothers of infants 12 months to 24 months out of the targeted 150 mothers of infants aged 6 to 24 months.

### Implementers of the study

All study phases were done by using a participatory approach, which was conducted by the trained health care workers (HCWs) of the rural village health unit and the village promoters (VPs) of the community-based association of the village under the supervision of the study team. The implementers were social workers; who were insiders, lived in the community, and understood its traditions, served as key players for the motivational messages and behavioral changes of weaning practices and as agents of change of the faulty practices.

All the implementers received training on the safe practices for healthy weaning. The implementers were targeted through a four-hour training lesson about healthy weaning practices to transfer the knowledge and skills to the mothers for sustainable health care.

### Intervention phase

This phase was the most important phase as its numerous implemented activities in the community aimed to provide knowledge about the targeted healthy nutritional behaviors for promoting and enhancing healthy weaning behavioral changes.

The interventions were based on the use of *communication for behavioral development approach (C4D)*.

### C4D Approach used for delivering the interventions

C4D was applied by the National Research Center team under the Slogan “Enjoy meals like a baby” meaning: eating nutritious and varied options through age-appropriate introduction and diversification of nutrient-rich complementary foods.

***C4D*** focused on five key behaviors’ concept to achieve the change:

1. **Perceived important change:** “That my baby is fed like a baby”
2. **Claimed behavioral change:** “I feed my baby like a baby”;
3. **Claimed intention of behavioral change: “**Making sure that your baby is fed like a baby”.
4. **How likely are you to start implementing the change?**
5. **Trust and advocate for behavioral change; the mother was asked: “**How likely would you recommend to any one near you “e.g. a relative or a neighbor” to start Making sure that their baby is fed like a baby”

### Intervention delivering process

Participants were targeted through weekly sessions related to healthy weaning and basic nutrition elements which were delivered to increase the knowledge of the mothers about healthy food (vegetables and fruits, proteins, carbohydrates, vitamins, minerals, milk & dairy products, and water). The mothers were taught, when to start weaning, continue breastfeeding till the age of 2 years, how to prepare a healthy nutritious meal for the proper age (quantity, consistency, and type). Hygiene and food safety were stressed on wash fresh fruits and vegetables before eating and keep food covered. Several nutritious meals were prepared and fed to their children to see how they liked it.

**Fig. 1:**
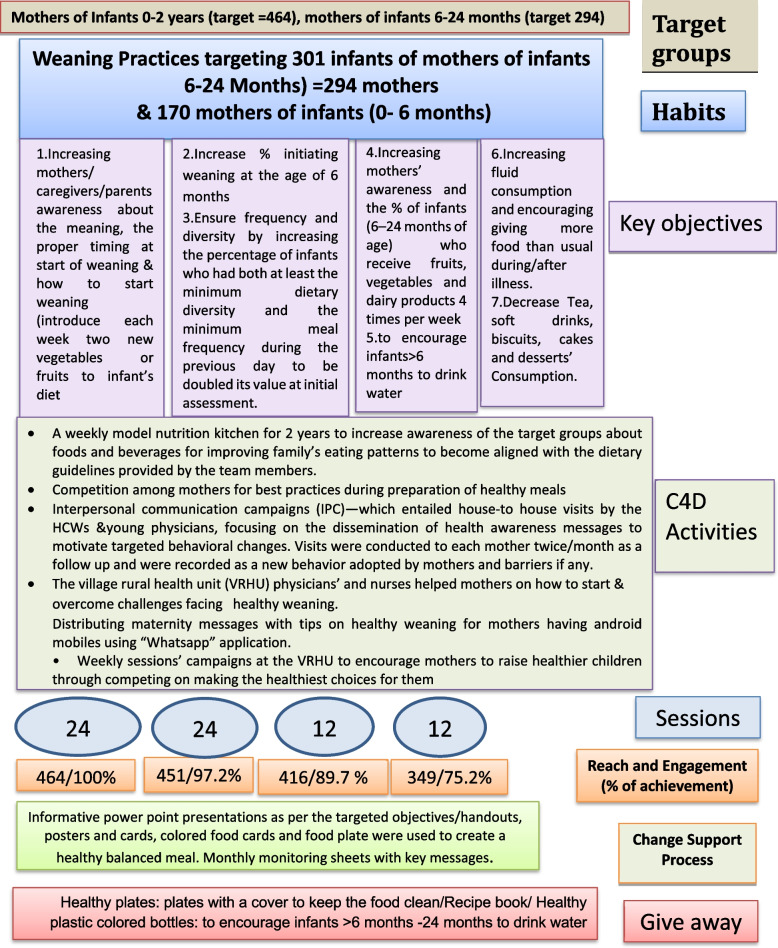
Flow diagram of the educational interventions’ messages, reach, engagement, tools and give-away using C4D approach

Eight messages were delivered over 12 months with new messages changed every three months. (3 messages were covered in the first session, 2 in the second, 4 in the third and 2 in the fourth along a month, meaning that repeating the same messages occurred every 3 months and that we had 4 rounds for the same messages along 20 months of implementation). The educational interventions: behavioral objectives, activities, reach, engagement, tools and give-away using the C4D approach were illustrated in Fig. 1.

### The key performance indicators for measuring the outcome of the intervention (assessment and evaluation phase)

To measure the intervention effectiveness, mothers and their infants were assessed and evaluated by the same structured questionnaires to test weaning practices and infant’s growth pre- and post-interventions. The whole intervention process took 24 months of activation.

Changes in the weaning practices as a result of the use of C4D approach were measured through; ***first*** comparing the changes in weaning practices resulting from the interventions between the interventional village and the control village, ***second*** evaluating the change of the weaning practices as well as the children's growth pre-and post-intervention within the interventional village considering that the study group served as self-control.

### Core indicators

1. *Introduction of solid, semi-solid or soft foods*
2. *Minimum dietary diversity*
3. *Minimum meal frequency*
4. *Minimum acceptable diet*
5. *Consumption of iron-rich foods*

### Core indicators definitions

The core indicators for assessing infant and young child feeding practices were defined according to the World Health Organization, 2008 [26] as follow:

1. *Introduction of solid, semi-solid or soft foods*: It is the proportion of infants 6–8 months of age who received solid, semi-solid, or soft foods during the previous day out of all infants aged 6–8 months.
2. *Minimum dietary diversity****:*** It is the proportion of children 6– < 24 months of age who received foods from 4 or more food groups during the previous day out of all infants aged 6- < 24 months of age. To calculate a value for this indicator, ***a seven-food group score variable*** was used for calculation, with an added point if any food in the group was consumed: grains, roots and tubers, legumes and nuts, dairy products (milk, yogurt, cheese), flesh foods (meat, fish, poultry and liver/organ meats), eggs, vitamin-A rich fruits and vegetables, and other fruits and vegetables.
3. *Minimum meal frequency*: It is the proportion of breastfed and non-breastfed children 6– < 24 months of age who received solid, semi-solid, or soft foods (but also including milk feeds for non-breastfed children) the minimum number of times or more during the previous day out of all breastfed infants aged 6- < 24 months of age and non-breastfed. To calculate a value for this indicator, combine the two numerators shown above, and the two denominators). This indicator summarizes several practices; frequency of feeding of solid, semi-solid, and soft foods of yesterday.
  - For breastfed children, the minimum number of times varies with age (2 times if 6–8 months and 3 times if 9– < 24 months).
  - For non-breastfed children the minimum number of times does not vary by age (4 times for all children 6– < 24 months).
4. *Minimum acceptable diet*: It is the proportion of children 6– < 24 months of age who received a minimum acceptable diet (apart from breast milk) during the previous day out of all children aged 6- < 24 months of age (breastfed and non-breastfed infants). The calculation of this indicator involves for the numerator combining breastfed infants and non-breastfed infants 6–– < 24 months of age who had at least the minimum dietary diversity and the minimum meal frequency during the previous day. The denominator involves both the breastfed and non-breastfed infants 6– < 24 months of age.
5. *Consumption of iron-rich foods*: It is the proportion of children 6– < 24 months of age who received an iron-rich food or iron-fortified food that is specially designed for infants and young children, or that is fortified in the home during the previous day out of all children aged 6- < 24 months of age.

### Dietary pattern assessment

For infants aged 12 to <24 months, the intake of different diversity of *nutrient-rich foods* (This includes: vegetables, fruits and nutrient-rich sources of protein and dairy) versus *calorie dense foods* with low levels of nutrients was compared for weekly consumption. The diversity of nutrient-rich foods eaten per week was measured as an indication of the weekly share of the stomach [27]. The weekly share of the stomach is to measure the percent intake of the six categories of the food pyramid along one week of consumption (Carbs/Grains, Dairy, Meat/ beans, fruits, vegetables, and snacks including sweets). An increase in the number of types of nutrient-rich foods overall and per food group that was eaten per week is considered an indication of a good share of the stomach from all the plates eaten every day.

### Setting targets as a results of the interventions

According to the assessment results, the target objectives were set to be realistic and achievable as follows:

For any indicator whose measure was 25% or less, the target was set to reach 50%. For any indicator whose measure was in the range of 25- 50%, the target was set to reach 75%. For any indicator whose measure was > 50% and < 75%, the target was set to reach 70–80%. For any indicator whose measure was > 75% the target was at least to sustain the indicator.

### Tools used for the assessment and evaluation phases

Participant *in-depth interviews* and *formative research* were used to collect data from studied women. The *in-depth interviews* focused on listening to the participants about problems they met in their previous experience and offering the best solution for each.

Through the *in-depth interview*, the faulty behaviors related to the weaning practices and their dominance were prioritized including: ways to introduce solid foods for infants ≥ 6 months of age, types and ways of preparing their complementary foods, and practices of caregivers on feeding infants when sick. The *in-depth interview* helped to identify also the key social and environmental contextual determinants of these faulty behaviors' practice.

The *formative research* was used to identify awareness and perception of proper weaning practices, the behaviors to be targeted and overall outcome goals in relation to when to start weaning, the proper food for age (type, consistency, and frequency), and which foods and drinks to be avoided at this age; assessing the factors that motivate and deter women to practice healthy weaning.

***A well-structured questionnaire*** was applied to the target groups through an interview to collect the nutritional data. The Centers for Disease Control (CDC) Infant Feeding Practices Questionnaire [27] was used to measure feeding practices throughout the first two years of life.

As part of the study, each mother received a number of self-report questionnaires at baseline, then monthly during a one-year follow-up. The daily dietary intake of infants at their first and second year of age was taken and analyzed for calculating the mean change of nutrients intake and their percent out of the Daily Recommended Intake(DRI) [28, 29].

***Growth assessment*** was done *to assess the prevalence of infant malnutrition in the village; overweight/obesity and stunting for infants aged* 6- < 24 months. The data of the included children were for those who fulfilled the inclusion criteria. Each of these children was measured twice; prior to the intervention and one year after. Out of 247 infants (119 males, 128 females) who were measured before the intervention, 180 children (72 males, 108 females) were included in the study whose mothers attended the activation process as well as who completed their growth assessment prior and after the intervention.

The following anthropometric measurements were performed:

*Bodyweight* was determined to the nearest 0.01 kg using a “***Seca Scale Balance***” for infants, with the infants wearing minimal clothes and with no shoes. *(Seca Balance Beam Scale Model 700, Seca Deutschland Medical Scales and Measuring Systems, secagmbh & co. kg., Hamburg, Germany).*

*Body length* was measured to the nearest 0.1 cm using a *Holtain portable anthropometer* and *Harpenden Infantometer*.

*Body circumferences* including head, neck, mid-upper arm, chest, waist, hip, mid-thigh, and calf circumferences using a flexible, non-stretchable tape were measured and approximated to the nearest 0.1 cm.

All anthropometric measurements were taken by using standard anthropometric protocols and standardized instruments. To allow a comparison of growth with the WHO and growth curves, z scores (WAZ, HAZ, and WHZ) were calculated for each infant. They were measured, following the recommendations of the “*International Biological Program”* [30, 31].

*Infant Body Mass Index (BMI)* was calculated: weight (in kilograms) divided by height (length) (in meters squared)**.** The infant’s BMI percentile was calculated for age and sex based on the Egyptian Growth Reference Charts [32].

The standard deviation score (Z score); to exclude the effect of age; was calculated for the weight (WAZ), height (HAZ), and BMI (BMI-Z) according to the Egyptian Growth Curve [33] by the following equation:

$$Z\;score=\frac{individual's\;variable\;-\;mean\;value\;of\;reference\;population}{SD\;of\;the\;reference\;population}$$

*Body composition* was measured using a body composition analyzer (Computerized Holtain Body Composition Analyzer -Holtain's BCA, Holtain ltd., UK, Wales, Crosswell No.646512). It displays Fat-free mass (FFM), Fat mass (FM) and Fat %, Total Body Water (TBW), and Basal Metabolic rate (BMR).

### Statistical analysis

Data were analyzed using Statistical Package for Social Sciences (SPSS) version 21.0 software. All data were represented by mean (SD) and percentages and comparisons between the pre and post interventions and between the intervention village and the control village were done using Chi square to study the pattern of distribution of the studied parameters and Z test was used between the two proportions. Unpaired and paired t tests were used to compare the differences between two means for the post intervention with the control and the pre and post interventions respectively. Probability values (P) of less than 0.05 were regarded as statistically significant [32]

The nutrients intake was analyzed for their macro, micro and elements content and compared with their DRI [33]

## Results

The interventional weaning practices targeted mothers of infants aged 6- < 24 months (*n* = 294) of 464 mother of infants from birth till 24 months. The interventions were delivered in the form of weekly sessions; concerning proper healthy weaning and basic nutrition education sessions, to increase the knowledge of the mothers about healthy food (vegetables and fruits, proteins, carbohydrates, vitamins, minerals, milk & dairy products, and water). The sampled mothers for evaluating the impact of the activities from both the intervention and the control villages were amounted to a total of 200 mothers of infants aged 0 to 24 months.

In the current study, it is apparent from the Table 1, that there was marked and significant improvement of the awareness indicators as a result of the intervention and between post-interventional and control villages (*P* = 0.001). Interestingly, the percentage of the mothers who knew the meaning (91%) and the time of starting the weaning at 6–8 months (100%), both increased strikingly in the intervention villages because of the intervention.

**Table 1:** Comparison of weaning awareness and practices Indicators between intervention and control villages

Indicators	Intervention village		Control village	*P* value of Z test Pre vs Post Intervention	*P* value of Z test Post Intervention vs Control
	Pre- intervention	Post- intervention			
Awareness Indicators of mothers					
	*N* = 200	*N* = 200	*N* = 200		
Know the meaning of weaning	117(58.5)	182(91.0)	110(55.0)	< 0.001^**^	< 0.001^**^
Know the exact time to start weaning (at 6–8 months)	120 (60.0)	200(100.0)	112(56.0)	< 0.001^**^	< 0.001^**^
Know how to start weaning: (Start with small amount of food then increase gradually over 2–3 months	126(63.0)	194(97.0)	130(65.0)	< 0.001^**^	< 0.001^**^
Intention to start weaning at 6 to 8 months	81 (40.5)	172 (86.0)	86 (43.0)	< 0.001^**^	< 0.001^**^
Practices Indicators for mothers of infants > 6 months-24 months					
	*N* = 150	*N* = 150	*N* = 150		
Gradual weaning (Started with small amount of food then increase gradually over 2–3 months)	93 (62)	135(90.0)	85(56.7)	< 0.001^**^	< 0.001^**^
Daily consumption of unhealthy food (24 h):					
•French fries or chips	126 (84)	102 (68)	129 (86)	< 0.001^**^	< 0.001^**^
•Biscuits	122(81.3)	51 (34)	126(84.0)	< 0.01^**^	< 0.01^**^
•desserts (oriental- sugary cookies- cake)	104(69.3)	60 (40)	96(64.0)	< 0.001^**^	< 0.001^**^
•Chocolate	93 (62)	38 (25.3)	84 (56.0)	< 0.001^**^	< 0.001^**^
•Ice cream	73 (48.70)	39 (26)	70 (46.7)	< 0.001^**^	< 0.001^**^
*P* value of X^2^	< 0.001^a**^ < 0.001^b**^				
Daily consumption of unhealthy liquid:					
Canned juice	128(85.3)	47(31.3)	131(87.3)	< 0.001^**^	< 0.001^**^
Tea	140(93.3)	75(50.0)	135(90.0)	< 0.001^**^	< 0.001^**^
Soft drinks	110(73.3)	21(14.0)	105(70.0)	< 0.001^**^	< 0.001^**^
*P* value of X^2^	< 0.001^a**^ < 0.001^b**^				
Weekly consumption of different food category (Share of Stomach) Ʊψ					
Nutrient rich food (Vegetables, Fruits, Dairyproducts, Meat& Legume)	62%	76%	57%	0.016^*^	0.04^*^
Energy dense food (Chips, Sweet, pastries)	16%	2%	18%		
Carbs, Grains	22%	22%	25%		
Breastfeeding during illness					
Increasing fluids	71(47.3)	116(77.3)	56(37.3)	< 0.001^**^	< 0.001^**^
Increasing breastfeeding	59(39.3)	85(56.7)	53(35.3)	0.003^**^	< 0.001^**^
Feeding your son variety of light meals	38(25.3)	48(32.0)	35(23.3)	0.201	0.093
Encourage your son to eat more food	24(16.0)	8(5.3)	28(18.7)	0.003^**^	< 0.001^**^
Stop breastfeeding	7(4.7)	0(0.0)	3(2.0)	0.007^**^	0.082
*P* value of X^2^	< 0.001^a**^ < 0.001^b**^				
Breastfeeding after illness					
Increasing fluids	26(17.3)	79(52.7)	33(22.0)	< 0.001^**^	< 0.001^**^
Increasing breastfeeding	53(35.3)	84(56.0)	48(32.0)	< 0.001^**^	< 0.001^**^
Feeding your son variety of light meals	48(32.0)	81(54.0)	45(30.0)	< 0.001^**^	< 0.001^**^
Encourage your son to eat more food	45(30.0)	20(13.3)	57(38.0)	< 0.001^**^	< 0.001^**^
*P* value of X^2^	< 0.001^a**^ < 0.001^b**^				

^*^significant < 0.05, **highly significant < 0.01, ^a^ *P* value of X^2^ between before and after interventions, ^b^ *P* value of X^2^ between after interventions and control, Ʊ % was calculated for infants aged 12–23 months (*n* = 100), ψ *P* values for the difference between the nutrient vs energy rich food

Moreover, the faulty weaning practices’ indicators among mothers of infants aged 6- < 24 months showed remarkable and significant improvement as a result of the intervention and between post-interventional and control villages (*P* = 0.001).

The most striking indicators were those of consumption of faulty food and liquids which showed dramatic improvement as in the reduction of the percentage of consumption of drinking tea from 93 to 50%, as well as canned juice from 85% to 31.3%.

There was also a clear and significant trend of increasing in all indicators related to feeding both during and after illness among infants aged 6- < 24 months, especially those concerning amounts of fluid and breastfeeding taken (from 47 to 77% and from 39 to 57% respectively) during illness (from 17 to 53% and from 36 to 56% respectively) and after illness, as a result of the intervention and between post-interventional and control villages (P < 0.01).

When we assessed the impact of the intervention on different weaning practices indicators among participants, the outcome of interest was *the remarkable increase* in the percentage of our targeted objectives, exceeding our expectations with highly significant difference between pre- and post-interventional village and between post-interventional and control villages (*P* = 0.001).

Unfortunately, some other indicators that were improved but couldn’t reach the percentage of our targeted objectives because of interventions are the "Minimum Dietary Diversity” (reached 32% only) and the minimum acceptable diet (reached 22.0% only).

This finding indicates that these two indicators need more attention during future interventions (Table 2).

**Table 2:** Impact of the C4D interventions on the target versus achievements of weaning practices’ indicators

Indicators	Intervention village			Control village *N* = 150	*P* value of Z test Pre vs Post Intervention	*P* value of Z test Post Intervention vs Control
	**Pre- intervention *N* = 150**	**Target Objectives as a result of the interventions**	**Post- intervention *N* = 150**			
**Introduction of Complementary Foods after 5 months of age**	55 (36.7)	**Need to be motivated** to reach 75%	123(82.0)	62(41.3)	< 0.001^**^	< 0.001^**^
**Advocated for Introduction of Complementary Foods after 5 months of age**	38 (25.3)	**Need to be motivated** to reach 50% -75%	101 (67.3)	43 (28.7)	< 0.001^**^	< 0.001^**^
**Minimum Dietary Diversity (receive food from 4 or more food groups)**	12(8.0)	**Need to be motivated** to reach 50%	48(32.0)	8(5.3)	< 0.001^**^	< 0.001^**^
**Minimum Meal Frequency (3 times if 6–8 months and 4 times if 9–23 months)**	68(45.3)	**Need to be motivated** to reach 50% -70%	101(67.3)	71(47.3)	< 0.001^**^	< 0.001^**^
**Minimum Acceptable Diet**	11(7.3)	**Need to be motivated** to reach 50%	33(22.0)	6(4.0)	0.004^**^	< 0.001^**^
**Dietary habits**						
**Consumption of milk (3 cups/day)Ʊ**	32 (32)	**Need to be motivated** to reach 75%	52 (52)	27 (27)	< 0.001^**^	< 0.001^**^
**Consumption of water (4 cups/day) Ʊ**	36 (36)	**Need to be motivated** to reach 75%	65 (65)	39 (39)	< 0.001^**^	< 0.001^**^
**Consumption of iron-rich or iron-fortified foods**	102(68.0)	**Need to be motivated** to reach 70%—80%	123(82.0)	88 (58.6)	0.002^**^	< 0.001^**^
**Consumption of honey (one spoon/day)**	63(42.0)	**Need to be motivated** to reach 55% -60%	87(58.0)	57(38.0)	0.002^**^	< 0.001^**^
**Regular check up for infants health at health unit**	65(43.3)	**Need to be doubled** to reach 66–70%	107(71.3)	83(55.3)	< 0.001^**^	< 0.001^**^

^**a**^*P* value between before and after interventions, ^**b**^*P* value between after interventions and control

^*^significant < 0.05, **highly significant < 0.01, Ʊ % was calculated for infants aged 12–23 months (*n* = 100)

Although the percentages of the daily consumption of milk with average of 3 cups /day and water with an average of 4 cups/day for infants aged 1–2 years were nearly doubled as a result of the interventions (from 32 to 52% and from 36 to 65% respectively), yet they are still lagging behind the target (75%).

One of the most interesting outcomes of interventions was its impact on the mean change of the mean daily nutrients intake and the percentage of Dietary Reference Intake (DRI) for infants 1–2 years (Table 3). It is obvious that all the nutrients either macro or micronutrients consumed by the infants were lower than their DRI. The percentages of daily caloric intake delivered from fat, protein, and carbohydrates among all the studied subjects in relation to their total caloric intake showed that the calories derived from protein were low even after the interventions (8.86%). Calories derived from fat were within the standard range, while calories derived from carbohydrate intake reached the upper limit after the interventions (65.08%).

**Table 3:** Impact of C4D interventions on the mean change of the nutrients intake and their percent of Daily Recommended Intake(DRI) for 1-2 years infants

Nutrients intake	Intervention village				Control village *N* = 100		*P* value of Z test
	**Pre- intervention *N* = 100**		**Post- intervention *N* = 100**				
	**Mean ± S D**	**% DRIs**	**Mean ± S D**	**% DRIs**	**Mean ± S D**	**% DRIs**	
**Energy (Cal)**	776.81 ± 35.21	59.75%	886.71 ± 39.46	68.20%	765.79 ± 37.13	58.37%	0.001^a**^ 0.001^b**^
**Protein % (10–30% of total Calories)**	7.75%		8.86%		6.75%		
**Fat (25–35% of Total Calories)**	15.76		26.06%		14.57%		
**Carbohydrate (45–65% of Total Calories)**	76.49%		65.08%		78.68%		
**Vitamin A (µg)**	258.74 ± 61.20	86.24%	264.50 ± 83.19	88.17%	220.70 ± 40.12	73.57%	0.578^a^ 0.001^b**^
**Vitamin D (µg)**	7.85 ± 1.23	52.33%	9.95 ± 1.41	66.33%	6.01 ± 1.22	40.07%	0.001^a**^ 0.001^b**^
**Vitamin C (mg)**	6.04 ± 2.62	40.67%	10.94 ± 2.62	72.93%%	5.39 ± 1.03	35.93%	0.001^a**^ 0.001^b**^
**Thiamin (mg)**	0.53 ± 0.24	106%	0.54 ± 0.38	108%	0.35 ± 0.38	70%	0.824^a^ 0.001^b**^
**Riboflavin (mg)**	0.54 ± 0.21	108%	0.56 ± 0.23	112%	0.33 ± 0.23	66%	0.522^a^ 0.001^b**^
**Niacin (mg)**	7.18 ± 1.02	119%	7.22 ± 1.12	120%	4.42 ± 1.12	73.67%	0.792^a^ 0.001^b**^
**Folic acid (µg)**	132.10 ± 3.24	88.10%	136.12 ± 5.28	90.74%	116.02 ± 3.31	77.35%	0.001^a**^ 0.001^b**^
**Vitamin B6 (mg)**	0.56 ± 0.32	112%	0.57 ± 0.42	114.0%	0.30 ± 0.47	60%	0.850^a^ 0.001^b**^
**Vitamin B12 (µg)**	0.75 ± 0.24	83.33%	0.87 ± 0.27	96.67%	0.57 ± 0.18	63.33%	0.001^a**^ 0.001^b**^
**Calcium (mg)**	537.88 ± 21.11	76.84%	594.97 ± 32.41	84.99%	478.11 ± 30.43	68.30%	0.001^a**^ 0.001^b**^
**Iron (mg)**	5.19 ± 1.62	74.14%	6.39 ± 1.62	91.29%	4.01 ± 1.20	57.29%	0.001^a**^ 0.001^b**^
**Zinc (mg)**	2.08 ± 1.51	69.33%	2.61 ± 1.31	87.0%	2.01 ± 1.20	67%	0.001^a**^ 0.001^b**^

^a^*P* value between before and after interventions, ^b^*P* value between after interventions and control

^*^significant < 0.05, **highly significant < 0.01

Moreover, data show a severe deficiency of some important micronutrients such as vitamins D & C and minerals like Ca, Iron & Zinc prior to the intervention and for the control group. Highly significant improvement after the interventions has led to an increase in the intake of the amount of these nutrients.

The impact of the interventions for proper weaning practices on the infants’ growth status was presented in Table 4. The frequency distribution of the sample according to Weight-for-age Z score (>—2 SD) as an indicator for underweight and Height-for-age Z score (>—2 SD) as an indicator for stunting showed that the recorded underweight and stunting among females over males disappeared and improved significantly as a result of the interventions (P < 0.01). On the other hand, the high percentage of wasting (Weight-for-Height Z score > -2 SD) among the males over the females disappeared significantly as a result of the interventions (P < 0.01). At the same time, the observed obesity disappeared completely among both males and females (P < 0.01). Moreover, the body composition parameters (FM, FFM, Fat % and TBW) were improved in favor of post-interventional phase’ growth parameters as a highly significant decrease was noticed regarding the mean Fat Mass and mean Fat% (From 5.47 ± 4.23 to 3.21 ± 1.47and from 32.36 ± 14.80 to 19.89 ± 6.77) respectively. In addition, a significant increase was seen for the TBW (from 10.15 to 14.99).

**Table 4:** Impact of C4D interventions on infants’ growth indices along one year of follow up

Variables	Pre intervention (*N* = 180)^a^ (Male = 72) (Female = 108)	post intervention (*N* = 180)^a^ (Male = 72) (Female = 108)	*P*
**WAZ Underweight**			
**Male**	13 18.1%	1216.7%	NS
**Female**	40 37.0%	1816.7%	< 0.01**
**Total**	53 29.4%	3016.7%	< 0.01**
**HAZ Stunting**			
**Male**	11.4%	6 8.3%	NS
**Female**	3 2.8%	0 0%	
**Total**	4 2.2%	6 3.3%	NS
**WHZ**			
**Wasting**			
**Male**	56.9%	00%	
**Female**	21.9%	00%	
**Total**	73.9%	00%	
**Overweight**			
**Male**	2737.5%	2433.3%	NS
**Female**	3431.5%	1211.1%	0.003**
**Total**	6133.8%	3620.0%	NS
**Obese**			
**Male**	1216.6%	00%	
**Female**	13 12.0%	00%	
**Total**	2513.9%	00%	
	**Mean ± SD**	**Mean + SD**	
**BMI (Kg/m**^**2**^**)**	8.60 ± 8.75	8.32 ± 8.52	0.237
**BMI percentiles**	72.66 ± 35.20	72.54 ± 31.34	0.986
**FFM**	10.16 ± 4.92	13.29 ± 2.79	0.003**
**FM**	5.47 ± 4.23	3.21 ± 1.47	0.009**
**Fat%**	32.36 ± 14.80	19.89 ± 6.77	0.000**
**TBW**	10.15 ± 6.49	14.99 ± 7.06	0.022*

^a^The only Data included for the analysis were for mothers who completed their attendance for the activation process and their nfants included prior to and after the intervention (180 infants out of 301 infants)

^*^significant < 0.05, **highly significant < 0.01, **¤** the tests are not conducted due to small sample sizes

*HAZ* Height-for-age Z score, *FFM* Fat free mass, *WHZ* Weight-for- Height Z score, *TBW* Total body water

## Discussion

The step of introducing food and maintaining intake of nutritious healthy foods could be assumed as a challenging cornerstone for parents especially in resource-poor settings which can lead to both stunted growth and delayed socioemotional development in infancy and early childhood [7, 34, 35]. Optimal feeding in the first 1000 days; from conception till the second year of life has the potential to shape individual health status during both childhood and adulthood [36] and even beyond; helping to reduce susceptibility to various infections like COVID-19 [37]. Therefore, providing adequate complementary feeding for infants 6– < 24 months old is mandatory to achieve the global target of reducing by 40% the number of stunted under five years by 2025 [38].

Globally, despite the fact that inappropriate complementary feeding practices remain a significant public health problem [39], yet, the information concerning complementary feeding is lacking. Consequently, this study was conducted aiming at providing an estimate for the indicators of the complementary feeding and evaluating the effectiveness of the provision of nutritional education intervention through the communication for development (C4D) approach over 2 years duration.

Implementing educational interventions through using the C4D approach at different community levels is widely recognized as effective in promoting public health practices in Egypt, especially in rural communities. These interventions have been used during the management of many health problems such as in managing children anemia [40] controlling endemic diseases like diabetes [41], HCV [42, 43], HBV [44, 45], renal diseases [46, 47], reducing maternal mortality [48], empowering of women [49], and improving sanitation [50, 51].

Our specific objectives were improving the weaning practices of mothers by introducing age-appropriate, diverse, nutrient-rich, and variety of food choices to achieve ‘‘*Feeding babies like babies and promoting infants’ growth*”. Referring to the WHO, 1998 [52], the complementary feeding should be timely, adequate, and appropriate, but despite its recommendations, the early introduction of complementary feeding remains common in both developed and developing countries [53, 54]. The current study revealed that as a result of 12 months’ engagement of C4D interventions of every mother enrolled the study, all participant mothers (100%) knew that starting weaning should be at 6–8 months. More than two-thirds of the mothers became very interested and started to delay weaning till the age of 6 months, increasing their introduction to timely complementary feeding from 36.7% to 82% of the infants aged 6 to < 24 months. The same result was reported by Edward and his colleagues, 2013 [55] on African-American mothers, showing a greater benefit of the intervention in delaying the start of the introduction of complementary feeding. Moreover, with our C4D interventions, the percentage of mothers who suddenly started weaning showed a marked decrease from 38% to reach 10%. This agreed with an Indian study, in which nearly half of the mothers gradually started weaning of their infants [56].

The world health organization (WHO, 2008) [26] has developed eight-core and seven optional indicators to monitor and promote adequate healthy infant feeding practices. The current study revealed that the minimum meal frequency indicator as a measure of the quantity dimension improved significantly and achieved the target (67.3%). The daily frequency of meals recommended for an infant depends on the amount of energy that he/she needs, the amount of food that he/she can eat per meal and the energy density of the food served [57]. Meanwhile, this indicator was low when compared to findings from Northern Ethiopia which was 82% [58]. The difference might be referred to low maternal educational status about optimal infant feeding practices in our study area.

On the other hand, the minimum dietary diversity indicator as a measure of the quality dimension, although improved, but still needs more attention because less than 40% of infants aged 6- < 24 months received food from 4 groups or more at a time as detected in post-interventional village evaluation. This finding was in accordance with Victor and his colleagues, 2014 [57] study in Tanzania. In fact, the low level of the minimum dietary diversity indicator affected the minimum acceptable diet indicator (combined both four or more food groups and minimum meal frequency indicators) which showed improvement to less than 25% of the targeted infants. This was relatively higher when compared to findings of other studies from southern Ethiopia [59]. UNICEF, 2019 [22], stated that in many countries only a few children obtain nutritionally appropriate complementary foods. Worldwide, the percentage of children who are not fed any fruits or vegetables is 44%, in addition, less than a fourth of infants 6– < 24 months of age meet the criteria of dietary diversity and feeding frequency that are appropriate for their age. UNICEF, 2019 [22] and WHO 2018 [5], both recommend that infants aged 6– < 24 months should eat at least (5–8) food groups with a range of nutrients to ensure normal healthy growth. In fact, the minimum acceptable diet indicator is considered as a core indicator to assess the level of appropriate complementary feeding.

In the present study**,** we reported that C4D interventions achieved remarkable significant improvement in the reduction of faulty weaning practices among mothers of infants aged 6- < 24 months) with increased nutrient-rich food (Vegetables, Fruits, Dairy products, Meat & Legume), and decreased energy-dense food (French fries, Chips, Sweet, pastries) which significantly doubled its value as a result of C4D interventions. Meanwhile, mothers could not reach the desired target of combining four or more nutrient-rich foods together. Besides the awareness issues, economic reasons, which are difficult to correct, may be responsible for not achieving this goal. Nevertheless, it is essential to raise the awareness of the community regarding the optimal complementary feeding practices, with special attention to the quality of feeding. Furthermore, the consumption of milk, iron-fortified food, and intake of fluids during and after illness among children 6- < 24 months of age increased.

On the other hand, there are still faulty dietary habits although significantly decreased, yet they need more focusing for minimizing them. On top of these was the consumption of the French fries and chips. Also, tea consumption (tea added to milk especially in the morning) was reported to be offered by half of the mothers to their infants to get more alert. In addition, most of the mothers found it very difficult to decrease offering sweets to their children.

One of the most interesting outcomes of C4D interventions was its impact on the mean change of the nutrients intake and the percentage of DRI for children 1–2 years. However, although the selected villages (intervention and the control one) have comparable socioeconomic characteristics with powerful and active well-organized community-based associations, it was noticed that for almost half of the vitamins and minerals assessed, there were significant differences between the baseline values in the intervention village and the values in the control village. We have realized that the local rural health unit in the control village was under construction which may be the explanation behind such a significant difference. The governmental local health units are used to provide nutritional and health services for the villagers. Apart from this, the significant improvement for the majority of nutrients as a result of the provided intervention in the intervention village is considered an asset for the success of the use of the C4D approach. The remarkable improvement of the majority of the indicators proved the success of C4D interventions.

Worldwide, in 2019, it has been estimated that 144 million children under 5 were stunted, 47 million were wasted, and 38.3 million were overweight or obese [11]. Black and his colleagues, 2008 [3] in their review***,*** specified receiving suboptimum complementary feeding to be a causal factor of stunting. In the current study, when interpreting the effect of interventions on children’s growth; sample aged 6- < 24 months through longitudinal study till aged 18–36 months, females recorded significantly marked improvement in the percentage of underweight (WAZ) and with the disappearance of stunting (HAZ scores). This could be related to cultural issues that are widespread in rural Egyptian regions. Unfortunately, families usually take care more of boys than girls, so girls received less care regarding their nutrition aggravating the problem of being underweight and stunted girls. Thanks to our C4D approach, follow-up of cases with nutritional supplements improved the underweight and stunting problems among girls indicating that targeting communities should be cultural-based. Basically, the growth rate is faster in malnourished girls due to catch-up phenomena than in boys of the same age which also played a role in the improvement. In addition, wasting, overweight and obesity (WHZ) disappeared among both sexes. These could be considered an excellent reflection of our research on the nutritional aspect of our growing children and the improvement of mothers^’^ behaviors. Similarly, in Australia, Lassi and his colleagues 2013 [60] stated in their review that educational interventions in complementary feeding had a significant effect on linear growth as evident by the remarkable increase of height gain, HAZ scores and WAZ associated with a considerable decrease in stunting. Other reviews published by Dewey and his coll0eagues, 2016 [61] in South Asia, and by Panjwani and Heidkamp, 2017 [62] in USA*, *reported a moderate impact on weight and linear growth*. *On the contrary, Arikpo and his colleagues,* 2015* [39] and Imdad and his colleagues, 2011 [63] in Pakistan, stated in their reviews that there were no effects of educational interventions on the prevalence of stunting, wasting, and underweight in children.

Preventive efforts should continue to focus on the 1000 days to prevent future overweight and obesity. Intrauterine, infant, and toddler periods are deemed to be possible crucial periods for programming long-term regulation of energy balance. [64–66]. Moreover, the early recognition of excessive weight gain relative to linear growth is essential to implementing policies of educational programs for girls during the childbearing period, pregnancy, and lactating mothers. Routine assessment of both weight and length in all children needs to become a mandatory clinical practice from birth. Behavioral changes such as encouraging exclusive breastfeeding delayed weaning, and promotion of appropriate complementary feeding practices are essential healthy nutritional habits.

## Conclusion

C4D allows undertaking formative research to identify gaps and resources available in the study locations. This allowed the intervention messages to be culturally appropriate and enhanced the acceptability and/or affordability of the interventions since most of the recommended foods were readily available in the intervention settings.

Targeting caregivers through the C4D approach have succeeded in providing them with the skilled support required for the provision of adequate nutrition for their infants and achieved an improvement of the timing and the process of complementary feeding.

Through the use of C4D, we have achieved the following: regression of early weaning practice, mothers learned to give proper food for age, offered their children fresher fruits and vegetables, increased consumption of water to up to 4 cups per day and increased milk intake. Dramatic reduction of consumption of canned juice and soft drinks, reduced consumption of faulty food and liquids, was observed. But still, mothers found it very difficult to decrease offering french fries or tea to their children.

Unfortunately, the "Minimum Dietary Diversity" and the "Minimum Acceptable Diet" indicators showed slight improvement, indicating required more focus to reach the target. The marked improvement on growth indices of infants is also an asset.

Although this study is a local one, it is the first to use the principles of the C4D approach in Egypt for changing faulty weaning practices to desirable ones. So this study provided a model for the effectiveness of the use of the C4D approach to achieve behavior changes through modifying negative ones and reinforcing optimizing behaviors. This model could be used and replicated by many resource-limited countries with a similar context. The use CAD approach if adopted by these countries will help achieve remarkable significant improvement in the reduction of faulty weaning practices. This is could be considered a strength of our study.

## Supplementary Information

**Additional file 1.**

## Data Availability

The datasets used during the current study are available from the corresponding author on reasonable request.

## Abbreviations

BMI: Body Mass Index
BMR: Basal Metabolic Rate
C4D: Communication for Behavioral Development
CBAs: Community-Based Associations
CDC: Centers for Disease Control
CPA: Charitable Patients Association in each village
DRI: Daily Recommended Intake
EBF: Exclusive Breast Feeding
FFM: Fat-Free Mass
FM: Fat mass
HAZ: Height-for-Age Z score
HCWs: Health Care Workers
KPIs: The Key Performance Indicators
P: Probability values
PHC: Primary Health Care
SD: Standard Deviation
SPSS: Statistical Package for Social Sciences
TBW: Total Body Water
VPs: Village Promoters
WAZ: Weight-for-Age Z score
WHO: World Health Organization
WHZ: Weight-for- Height Z score
