# Supplementary material for: Promoting weaning practices and growth of Egyptian infants by using communication for behavioral development approach
Source: BMC Pediatr. 2022 Dec 1;22:689. doi: 10.1186/s12887-022-03741-0 (PMC9713754; doi:10.1186/s12887-022-03741-0)
Supplement: Supplementary file 1 — Additional file 1. [file 12887_2022_3741_MOESM1_ESM.docx]

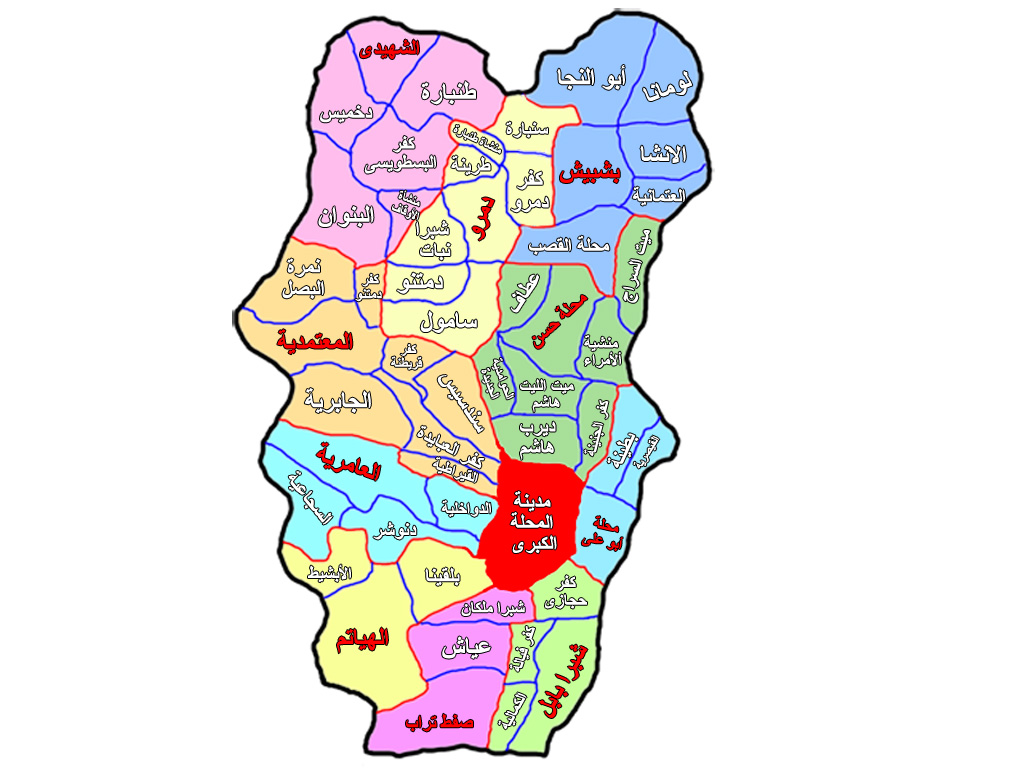


El Othmanyia village (intervention village)

Nemra El Basal (control village)

**Map concerning the site of the intervention and the control villages**

“According to the geographical distribution, both villages were selected from the same governorate and the same district to have the same sociodemographic status, but in different local village units (one in the east and the other in the west of El Mahallah El Kubra district) to prevent possible contamination so that real impact could be assessed.
